# Sustainable Triboelectric Nanogenerator from Abalone Shell Powder for Self-Powered Humidity Sensing

**DOI:** 10.3390/s25247584

**Published:** 2025-12-14

**Authors:** Yunsook Yang, Farhan Akhtar, Shahzad Iqbal, Muhammad Muqeet Rehman, Woo Young Kim

**Affiliations:** 1 Faculty of Applied Energy System, Department of Electronic Engineering, Jeju National University, Jeju 63243, Republic of Korea; yunsuk0001@jejunu.ac.kr (Y.Y.); farhanakhtar@stu.jejunu.ac.kr (F.A.); shahzadiqbal@stu.jejunu.ac.kr (S.I.); 2 Faculty of Electrical Engineering, Ghulam Ishaq Khan Institute of Engineering Sciences and Technology, Topi 23640, Pakistan

**Keywords:** abalone shell powder triboelectric nanogenerator (ASP-TENG), self-powered humidity sensor, sustainable circular electronics

## Abstract

Self-powered sensors are critically important for IoT, yet most rely on synthetic polymers that lack environmental sustainability. This work presents a triboelectric nanogenerator (TENG) made from marine biowaste which operates as both an energy generator and humidity sensor. Abalone shell powder (ASP) majorly composed of calcium carbonate (CaCO_3_) was used as its tribopositive layer in combination with polytetrafluoroethylene (PTFE) as tribonegative layer. The developed ASP-TENG device generated 410 V peak to peak open-circuit voltage (V_OC_) and 2.79 W·m^−2^ peak power density at an operating frequency of 4 Hz. These obtained results match or surpass existing biowaste-based TENGs. ASP-TENG efficiently worked as a self-powered humidity sensor because its output voltage decreased steadily from 410 V to 176 V in response to an increase in relative humidity (%RH) from 40% to 80% (decreases of 5.8 V for every 1%RH). The triboelectric charges become screened by water molecules that adsorb onto the porous CaCO_3_ surface which leads to faster leakage current. This work demonstrates a sustainable method to create TENGs with multiple functions while developing environmentally friendly sensing systems for environmental tracking and sustainable energy harvesting.

## 1. Introduction

The measurement of humidity plays a critical role in environmental monitoring systems, agricultural practices, healthcare facilities, and industrial production lines [1,2]. The operation of conventional sensors which use capacitive/resistive/impedemetric or optical/mass-sensitive principles depends on external power sources which restrict their sustainability [3,4,5]. The development of self-powered humidity sensors now exists as a solution for the present limitations of such sensors with the ability to generate their own power from environmental sources while performing sensing operations [6]. The development of TENG-based sensors could enable simultaneous energy harvesting and detection through their ability to transform surface charge variations caused by moisture into electrical signals [7]. Additionally, from the material perspective, the development of green sensing technologies continues through two new approaches which include electrochemical sensors (that use redox reactions) from humidity and ion-gradient sensors (that use ionic diffusion) [8]. The development of self-powered sensors faces two main problems: the production of unstable output signals and the inability of materials used in them to withstand long-term use. Researchers have dedicated their research to using humidity-sensitive materials by designing them for specific hydrophilic properties, porous structures, and functional groups to boost both charge transfer and moisture absorption capabilities.

The Internet of Things (IoT), smart infrastructure, and environmental monitoring systems require immediate development of autonomous sensors [9]. Battery-powered nodes require frequent maintenance, have short lifespans, and generate electronic waste (E-waste), undermining sustainability goals. Self-powered technologies now offer a promising solution for building distributed sensing networks because they can extract energy from their surrounding environments. TENGs are widely studied as a leading technology for converting low-frequency mechanical motion into electricity through contact electrification and electrostatic induction. Since their introduction in 2012, TENGs have evolved into versatile platforms for energy harvesting and signal sensing in applications such as wearables, medical devices, structural health monitoring, and environmental surveillance [10,11,12,13,14,15]. Their lightweight and flexible material properties make them ideal for operating distributed IoT nodes. The most prominent and well established modes of operations of TENGs include contact-separation mode [16], sliding mode [16], freestanding mode [17], single electrode TENG [18], and dual-electrode mode [19]. Moreover, the adsorption of moisture on tribo-surfaces leads to faster charge loss which decreases output performance and reduces energy harvesting efficiency. However, this challenge can be leveraged as an opportunity, since the same adsorption process could also enable humidity detection through optimized tribo-layer design for water-adsorption-based transduction. While humidity-sensitive TENGs have been reported, most rely on synthetic polymers or surface treatments, raising sustainability and E-waste concerns. A recent trend among researchers is to explore suitable biowaste materials for use as triboelectric layers to solve previous limitations [20,21,22,23,24,25,26,27,28]. This approach perfectly aligns with circular economy principles by repurposing waste materials into functional components for sustainable electronics. The development of TENG technology benefits from biogenic materials through several significant examples. Onion peel-based TENGs leverage the fibrous structure and dielectric properties of plant tissues to achieve voltages over 800 V and power densities up to 8 W·m^−2^ [22]. Flexible TENGs made from eggshell membranes show improved biocompatibility, while chitosan-based and cellulose-based devices demonstrated both biodegradability and environmentally friendly characteristics [16,24,27]. Marine shells, rich in calcium carbonate (CaCO_3_), are an abundant biowaste yet remain underexplored for TENG applications. The natural properties of seashells make them suitable for triboelectric charge production because they have a complex microstructure with a rough surface and contain two different crystalline phases of aragonite and calcite [21]. Research using scallop and clam shells has proven that seashell powders work as tribopositive layers for energy harvesting by producing 200 V output voltages and 0.95 W·m^−2^ power densities. Moreover, research teams have recently worked to develop active TENG-based humidity sensors which use engineered nanomaterials for operation. The combination of graphene oxide (GO) with graphene oxide nanoribbon (GONR) in TENGs produced monotonic RH responses with 0.5 V/%RH sensitivity and 6–8 s response times [29]. CNT-PDMS foam TENG sensors delivered 0.6 V/%RH sensitivity with rapid response and recovery [30]. The developed humidity-sensitive TENG systems proved the idea but required complicated manufacturing methods, costly materials, and lacked environmental sustainability. Existing research lacks strategies that could combine high energy output with reliable humidity sensing through using sustainable materials like marine shell waste, particularly abalone shells, which offer a dual-crystalline structure and superior strength over other mollusks.

Our study particularly addresses this gap by introducing a high-performance abalone shell powder-based triboelectric nanogenerator (ASP-TENG) device, using mechanically ground ASP as the tribopositive layer without chemical processing. Polytetrafluoroethylene (PTFE) was used as a tribonegative material of ASP-TENG to achieve better charge production and storage. The ASP-TENG achieved maximum power density at a 10 MΩ load, with its electrical output increasing linearly with contact area and motor frequency while maintaining stable operation. ASP-TENG operated as a self-powered humidity sensor, delivering high sensitivity of 6.03 V/%RH (surpassing existing TENG-based sensors) due to natural charge screening on its hydrophilic CaCO_3_ surface. This research transformed marine shell waste into sustainable smart infrastructure and a wearable microclimate sensor through a circular approach.

## 2. Materials and Methods

**Figure 1:**
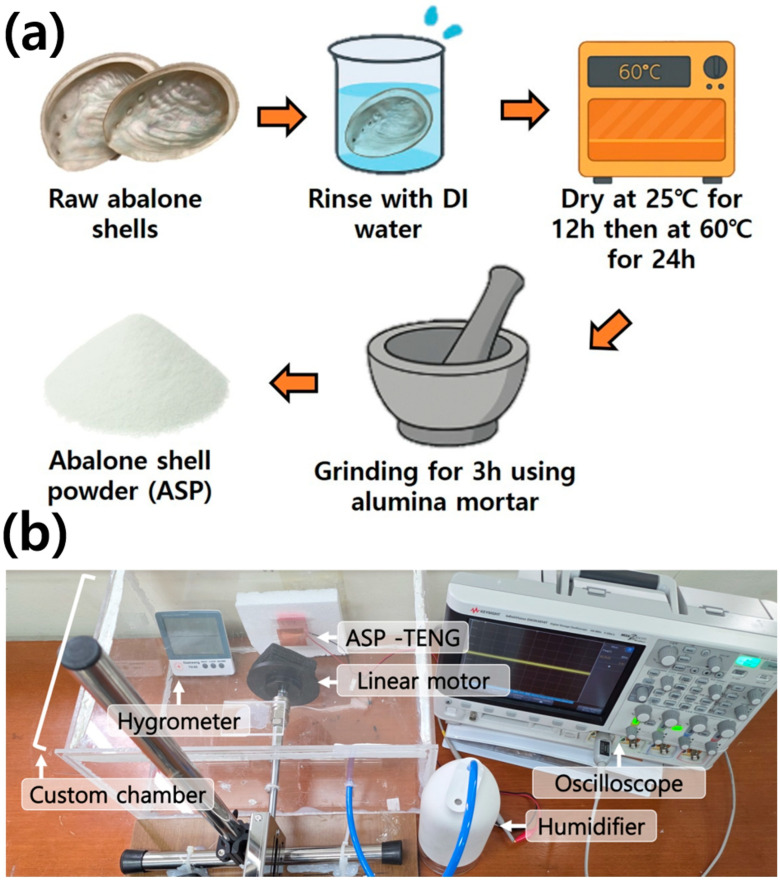
(**a**) Schematic diagram containing optical images of different steps involved in the preparation of ASP including the schematic diagram of the final developed ASP-TENG. (**b**) Optical photograph of experimental setup used to measure self-powered humidity sensitive response of developed ASP-TENG device with each part labeled.

The research team obtained abalone shells (*Haliotis* spp.) from seafood processing facilities before washing them with deionized water to eliminate salts and other organic substances. The shells underwent air drying at 25 °C for 12 h followed by oven drying at 60 °C for 24 h to remove all remaining moisture. The high-speed grinder processed the dried shells into ASP through mechanical grinding. No chemical treatments were applied during ASP purification to preserve the natural crystalline structure and surface chemistry, as shown in Figure 1a. The obtained ASP was then applied to a copper (Cu) electrode to create the tribopositive layer while PTFE served as the tribonegative layer. Both layers were attached to polyethylene terephthalate (PET) substrates to operate the developed ASP-TENG device in a vertical contact–separation mode. Devices of three different sizes were developed (2 × 2 cm^2^, 3 × 3 cm^2^ and 4 × 4 cm^2^), respectively, to study the effect of contact area on output performance.

We used a scanning electron microscope (SEM) (Phenom Pharos G2 from Thermo Fisher Scientific in Seoul, Republic of Korea) to study the surface characteristics of ASP while Energy Dispersive X-ray Spectroscopy (EDS) provided data of its elemental composition. The X-ray Diffraction (XRD) (Empyrean system from Malvern Panalytical, Malvern, Worcestershire, UK) operated from 20° to 60° (2θ) to detect traces of crystalline structure, phase composition, and lattice parameters in the ASP. Fourier Transform Infrared Spectroscopy (FTIR) (Alpha II from Bruker Optiks in Ettlingen, Germany) was also used to study functional groups across the 4000–500 cm^−1^ spectral range. Electrical characterization of the ASP-TENG was conducted using a linear motor (1–4 Hz) for vertical contact–separation tests to measure triboelectric output. Open-circuit voltage (V_OC_) was measured using an oscilloscope (Keysight DSOX3014T from Keysight Technologies, Santa Rosa, CA, USA) and short-circuit current (I_sc_) with a Keysight B2911A source meter (Keysight 2911A from Keysight Technologies, Santa Rosa, CA, USA). Load-dependent behavior of the developed ASP-TENG device was studied through resistance changes from 0.01 MΩ to 100 MΩ. Most importantly, we studied the effects of varying %RH on the developed ASP-TENG device by placing it in our customized controlled environmental chamber as shown in the optical picture of Figure 1b. Humidity was regulated in a sealed chamber using nozzles for humidification, nitrogen-based dehumidification, and linear motor insertion. The customized chamber housed the ASP-TENG device and a digital hygrometer (for real-time monitoring of %RH and temperature) inside the customized measurement chamber. Humidity was stepwise adjusted from 40% to 80% RH, stabilized before testing, and at each level the linear motor impacted the ASP-TENG while output voltage was recorded on a digital oscilloscope. Sensitivity was defined as the relative change in output per %RH change, response time as the duration to reach 90% of final output after a humidity shift, and recovery time as the time to return to 10% of baseline after %RH decreased.

## 3. Results

### 3.1. Micro-Structure and Composition Underpin High Surface Charge Generation

**Figure 2:**
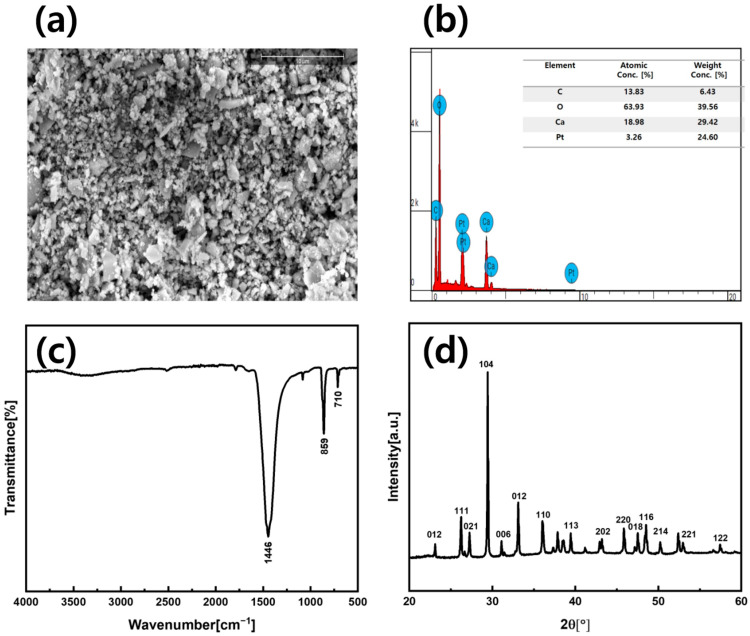
Material characterizations: (**a**) SEM image of ASP showing hierarchical roughness with micropores and asperities, enhancing triboelectric contact and moisture adsorption. (**b**) EDS spectrum confirming CaCO_3_ composition (Ca, C, O) with no contaminants, ensuring dielectric stability for TENG and humidity sensing. (**c**) FTIR spectrum displaying carbonate-specific bands, indicating preserved chemical structure for triboelectric performance and water interaction. (**d**) XRD pattern showing calcite and aragonite phases, contributing to charge-trapping sites and humidity-responsive surface energy.

The complete analysis proved ASP suitable for performing two specific functions of triboelectric energy harvesting and self-powered humidity sensing. SEM image (Figure 2a) exhibited ASP grains with irregular shapes, micro-pores, and pointed asperities, thus forming a hierarchical surface that enhanced charge transfer and moisture absorption. The EDS results (Figure 2b) demonstrated that ASP contained mainly Ca, C and O elements which matched CaCO_3_ composition without any foreign substances or impurities that would affect dielectric stability. The FTIR spectrum (Figure 2c) showed peaks at 1446 cm^−1^ (asymmetric stretching), 859 cm^−1^ (out-of-plane bending), and 710 cm^−1^ (in-plane bending), confirming carbonate groups and calcite/aragonite polymorphs. The FTIR spectrum showed peak splitting between 1400 and 1500 cm^−1^ which indicated the presence of amorphous calcium carbonate [31]. FTIR spectrum of ASP showed peaks at 1446, 859, and 710 cm^−1^, indicating hydroxyl and related functional groups typical of cellulose and pectin-rich materials. Hydroxyl groups were well-recognized electron-donating active sites and were considered a standard indicator of tribopositive behavior in biopolymeric materials. This chemical evidence strongly supported the suitability of ASP as a tribopositive layer for ASP-TENG fabrication, consistent with previously reported biowaste-based triboelectric materials in the literature. The X-ray Diffraction (XRD) pattern (Figure 2d) showed sharp peaks at 012, 111, 021, and 104, indicating high crystallinity and multiple polymorphs that enhanced dielectric properties, charge retention, and electron affinity [32]. The hydrophilic nature of carbonate groups and amorphous content in ASP enabled fast water absorption which led to sensitive and reliable humidity measurements. The material properties of ASP complemented each other to achieve maximum triboelectric output and high humidity sensitivity, making it suitable for self-powered sensing applications.

### 3.2. Output Scales with Excitation Frequency and Contact Area

**Figure 3:**
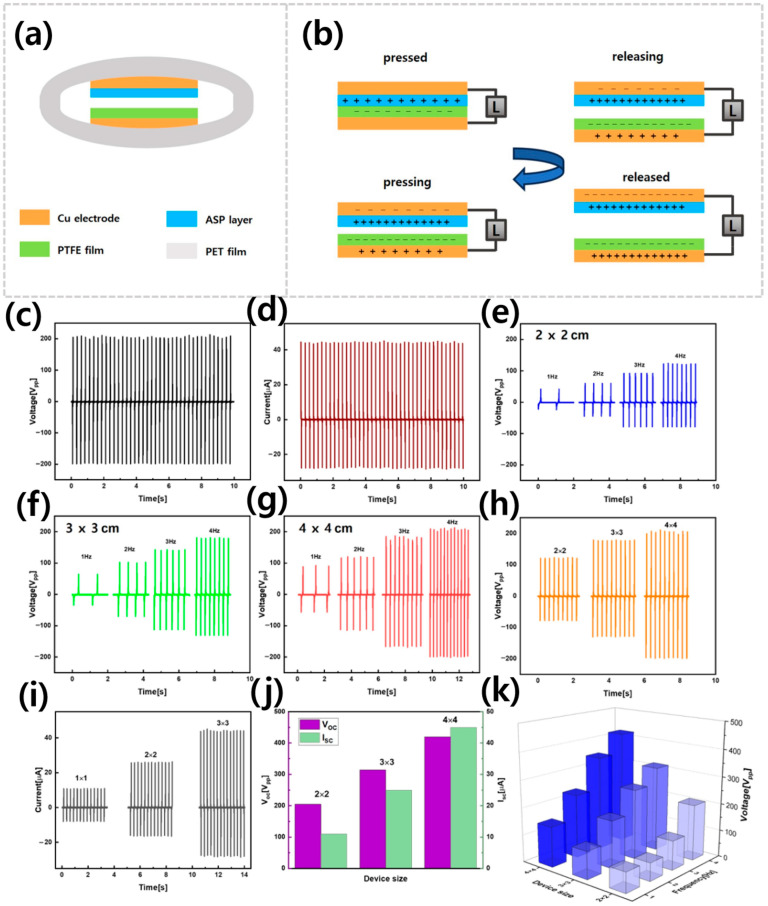
Energy harvesting results of ASP-TENG (**a**) Labeled schematic diagram of ASP-TENG, (**b**) Schematic diagram of contact–separation mode of operation for the developed ASP-TENG. (**c**) Highly stable AC output voltage. (**d**) Highly stable AC output current. (**e**–**g**) Effect of striking frequency on the electrical output of ASP-TENG with different device size. (**h**) Effect of varying contact area on the output voltage. (**i**) Effect of varying contact area on the output current. (**j**) 2D bar graph comparison of output voltage and output current with varying device size (**k**) 3D bar graph of electrical outputs of ASP-TENG with reference to operating frequency and device size.

Figure 3a shows the ASP-TENG schematic with labeled layers, while Figure 3b illustrates its standard contact–separation conduction process. The overall characterization setup (Figure 1b) transformed the mechanical energy of the linear motor into electrical power through triboelectrification and electrostatic induction processes. In contact–separation mode, the ASP and PTFE layers generated triboelectric charges upon full contact due to their contrasting electron affinities, creating a localized electric field. Trapped charges drove the electron flow through the circuit when both tribolayers separated, raising the potential difference to its peak value at maximum separation. During recontact, the potential dropped, driving electrons in the reverse direction to complete the cycle. This repetitive contact–separation process converted low-frequency mechanical energy into alternating electrical signals without requiring any external power source. The ASP-TENG produced its maximum open-circuit voltage (V_OC_) of 410 V (peak to peak) and short-circuit current (I_SC_) of 45 µA according to the experimental results shown in Figure 3c,d. The ASP-TENG device output performance improved when the operating frequency of the linear motor increased from 1 Hz to 4 Hz during vertical contact–separation operations (Figure 3e–g). The displacement current and total charge transfer per unit time also increased with a rise in operating frequency which resulted in higher output voltage and current. The output voltage and current measurements showed direct proportionality to the contact area size (Figure 3h–j) because larger interfaces enabled the storage of more surface charge (σ × A). Hence, the 4 × 4 cm^2^ device operated at 4 Hz produced the highest electrical output according to the 3D analysis presented in Figure 3k. These results of electrical output clearly demonstrated high surface charge density and strong electrostatic induction. The electrical output patterns matched the material properties that were previously measured through SEM, XRD, and FTIR tests. ASP-TENG maintained stable performance at high charge density across multiple cycles, owing to its structural and chemical properties suited for self-powered sensing.

### 3.3. Load Dependence and Peak Power Density

**Figure 4:**
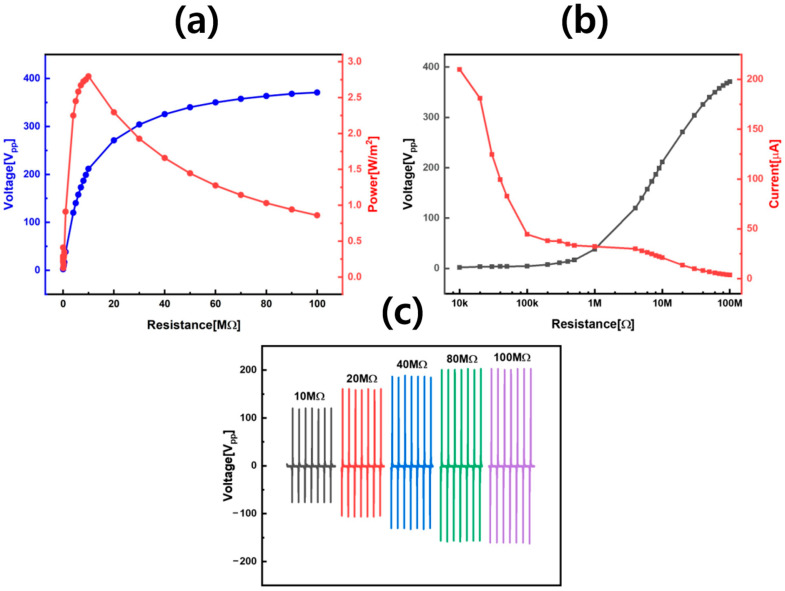
Load-dependent performance of ASP-TENG showing the effect of resistance on voltage, current, and power, and identifying optimal impedance for peak energy output. (**a**) Variation in output voltage and power density with load resistance (0.01–100 MΩ). Voltage increases while power peaks at 10 MΩ (2.79 W m^−2^), indicating optimal impedance matching for maximum energy transfer. (**b**) Variation in output voltage and current with load resistance. The non-linear I–V trend confirms the capacitive nature of ASP-TENG, governed by electrostatic induction rather than ohmic conduction. (**c**) Comparison of output voltages across different load conditions (10–100 MΩ). Higher resistance favors voltage amplification, critical for powering high-voltage, low-current sensors and storage systems.

The ASP-TENG showed distinct capacitive behavior when operated under different load resistance values between 0.01 MΩ and 100 MΩ as shown in Figure 4a–c. ASP-TENG output voltage increased while current decreased as load resistance rose from 0.01 MΩ to 100 MΩ (consistent with its dielectric properties). Electrostatic behavior of ASP-TENG was evident by its charge–voltage characteristics, revealing that higher resistance significantly enhanced voltage accumulation. The power density output reached its maximum value of 2.79 W m^−2^ when the 10 MΩ resistance was used as R_L_, which indicated the best impedance ratio for maximum energy transfer had reached this point. The identification of this maximum power transmission point could be helpful in enabling design engineers to create rectification and storage circuits for achieving optimal power management. I–V characteristics (Figure 4b) exhibited non-linear behavior, confirming that ASP-TENG operated as a capacitive source via electrostatic induction and Maxwell’s displacement current rather than Ohmic conduction. The selection of proper load conditions becomes even more important for achieving maximum energy conversion efficiency according to this important result. Figure 4c compared ASP-TENG output voltage across 10–100 MΩ loads, highlighting its scalability under varying resistance. The ASP-TENG device produced better output voltage when operated at high resistance values, making it suitable for applications that require high voltage and low current for sensor operation. Figure 4 shows that impedance tuning maximizes ASP-TENG performance for self-powered applications.

### 3.4. Self-Powered Humidity Sensing with Monotonic Response

**Figure 5:**
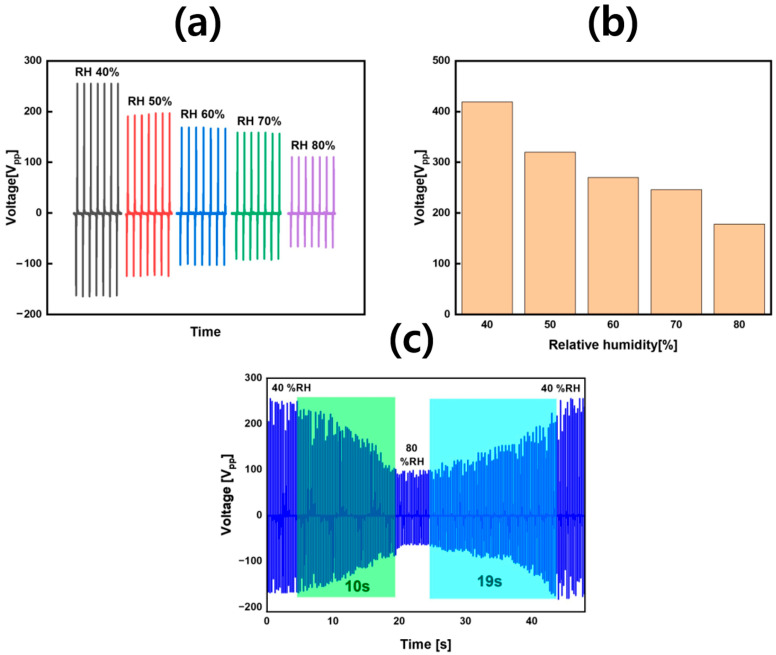
Humidity-dependent electrical response of ASP-TENG demonstrating its self-powered sensing capability and monotonic voltage decrease with increasing relative humidity (RH). (**a**) Continuous decline in peak-to-peak AC voltage from 410 V at 40% RH to 176 V at 80% RH, confirming stable and repeatable humidity-sensing behavior. (**b**) Bar graph representation of voltage response across RH levels (40–80%), highlighting monotonic trend and suitability for calibration in self-powered humidity sensors. (**c**) Reaction time (response and recovery) of the developed ASP-TENG for a humidity shift between 40% to 80% RH.

ASP-TENG output voltage dropped from 410 V to 176 V as humidity increased from 40% to 80% RH at 25 °C (Figure 5). The ASP-TENG sensor exhibited robust performance by delivering a consistent and single-directional response to varying relative humidity (%RH) while maintaining repeatability across multiple measurement cycles. Voltage-mode sensitivity of the ASP-TENG humidity sensor was calculated to be equal to 6.03 V/%RH by using the numerical values obtained from the results of Figure 5. We also measured the response and recovery time for our self-powered ASP-TENG-based humidity sensor and the values turned out to be 10/19 s for response/recovery time, respectively, as shown in Figure 5c. The humidity-sensing mechanism could be explained through referring to the material characterization results of the ASP surface shown in Figure 2. SEM analysis revealed hierarchical roughness and interconnected micro/mesopores on the ASP surface, providing abundant contact points for high charge density under dry conditions and enabling nanometric water film formation at elevated %RH. FTIR confirmed abundant carbonate groups in ASP, enabling its strong water adsorption through ionic and hydrogen bonding. Furthermore, XRD revealed calcite and aragonite phases in ASP, creating charge-trapping boundaries that ensured high baseline output. Rising %RH decreased V_OC_ as water adsorption formed a conductive layer that screened surface charges. The EDS results also showed that the ASP consisted only of Ca, C and O atoms, proving that the humidity response originated from the ASP/PTFE interface rather than external contaminants. ASP’s high crystallinity and polymorphic interfaces delivered strong low-RH performance and predictable humidity response. The dual functionality of ASP-TENG made it suitable for self-powered environmental monitoring systems because it operated as a sustainable material solution that followed the principles of circular economy. Furthermore, we have compared the performance of our developed ASP-TENG with other recently reported natural material based TENG devices in Table 1.

### 3.5. Practical Demonstration of ASP-TENG for Energy Harvesting and Sensing

**Figure 6:**
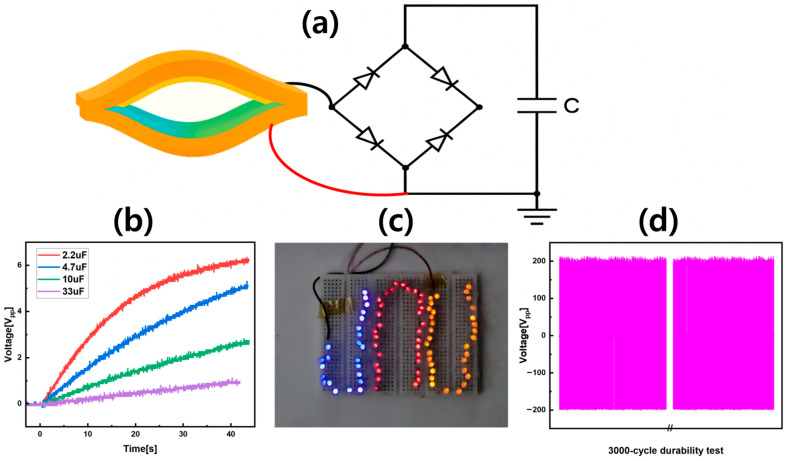
Practical demonstration of ASP-TENG for energy harvesting and sensing applications, including circuit integration, capacitor charging, LED powering, and long-term stability. (**a**) Schematic of ASP-TENG connected to a Wheatstone bridge for AC-to-DC conversion. (**b**) Charging curves for commercial capacitors (2.2–33 µF) powered by ASP-TENG. (**c**) Direct powering of multiple LEDs (blue, red, yellow) using harvested energy. (**d**) Repeatability test over 3000 cycles showing stable AC output voltage and mechanical durability.

The practical implementation of ASP-TENG in Figure 6 demonstrated its ability to operate as both an energy-harvesting device and a self-powered humidity sensor. The Wheatstone full-bridge rectifier schematic shown in Figure 6a enabled ASP-TENG to convert its AC electrical output into DC voltage which powered electronic components without requiring external bias. This system design proved battery-free operation capability and ultra-low-power electronics compatibility which made it appropriate for distributed sensing nodes and IoT applications. The ASP-TENG device in Figure 6b demonstrated its ability to store energy from mechanical motion by charging commercial capacitors with capacities from 2.2 µF to 33 µF. The device’s charging profile demonstrated its capability to deliver sufficient energy for short operational bursts—a critical requirement for autonomous sensors and wireless communication systems. The ASP-TENG device in Figure 6c demonstrates its ability to power several LEDs of different power ratings (blue, red, yellow) which proves its suitability for smart system applications that need real-time visual feedback and low-power signaling. The 3000-cycle repeatability test in Figure 6d proves that ASP-TENG maintains excellent mechanical durability and electrical stability for extended environmental monitoring deployment. Practical results aligned well with material characterizations as SEM revealed hierarchical roughness and porosity that boosted charge generation; XRD confirmed high crystallinity and polymorph diversity enhancing dielectric performance; and FTIR detected hydrophilic carbonate groups enabling humidity sensing without compromising energy harvesting. The combined properties of ASP-TENG enable it to operate as both a power source and sensing element, which simplified system design and reduced cost while using seashell waste to create sustainable products.

## 4. Conclusions

This work demonstrated an environmentally sustainable approach to fabricate multifunctional triboelectric nanogenerators (TENGs) by recycling abalone shell waste powder (ASP) into a high-performance device capable of dual functionality of energy harvesting and self-powered humidity sensing. The ASP-TENG leveraged the intrinsic properties of biogenic CaCO_3_, high crystallinity, polymorphic interfaces, and hydrophilic surface chemistry to deliver strong electrical output while providing a stable, monotonic %RH response without external bias. This study further established that biowaste is not only a viable material for triboelectric applications but also enables humidity-based charge screening to serve as a sensing mechanism rather than a limitation. Future work related to this work can include optimizing output performance through surface-energy modification and porosity engineering, paving the way for wireless humidity monitoring in smart buildings, IoT networks, and wearable microclimate sensors. By combining sustainability with advanced functionality, this research contributes to the development of next-generation self-powered systems aligned with circular economy principles.

## Figures and Tables

**Table 1:** Output performance comparison of developed ASP-TENG with other natural-material-based TENGs.

Natural Material	Other Material	Open Circuit Voltage (V)	Short Circuit Current (µA)	Power Density (mW/m^2)^	Application	Reference
Coconut husk	Kapton	14	0.05	3.5	Breathing pattern monitor	[33]
Aloe vera	PDMS	32	0.11	1.9	Self-powered finger monitoring sensor	[34]
Nopal powder	Polyimide	15.28	0.38	2.145	Smart motion sensing	[35]
Collagen	PVDF	118	0.0038	21.06	Light-up commercial LEDs	[36]
Diatom frustule-chitosan	FEP	150	0.86	15.7	Motion sensor	[37]
Korean seaweed	Edible silver leaf	23	0.315	2	Self-powered devices	[38]
Abalone shell powder (ASP)	PTFE	255	45	2790	Self-powered humidity sensing	This work

## Data Availability

The raw data supporting the conclusions of this article will be made available by the authors on request.
